# GV1001 reduces neurodegeneration and prolongs lifespan in 3xTg-AD mouse model through anti-aging effects

**DOI:** 10.18632/aging.205489

**Published:** 2024-01-31

**Authors:** Hyun-Hee Park, Hyuk Sung Kwon, Kyu-Yong Lee, Ye Eun Kim, Jeong-Woo Son, Na-Young Choi, Myung-Hoon Han, Dong Woo Park, Sangjae Kim, Seong-Ho Koh

**Affiliations:** 1 Department of Neurology, Hanyang University Guri Hospital, Gyeongchun-ro, Guri-si, Gyeonggi-do 11923, Korea; 2 Department of Translational Medicine, Hanyang University Graduate School of Biomedical Science and Engineering, Seoul 04763, Korea; 3 Department of Neurosurgery, Hanyang University Guri Hospital, Gyeongchun-ro, Guri-si, Gyeonggi-do 11923, Korea; 4 Department of Radiology, Hanyang University Guri Hospital, Gyeongchun-ro, Guri-si, Gyeonggi-do 11923, Korea; 5 Teloid Inc., Los Angeles, CA 90010, USA

**Keywords:** 3xTg-AD mice, GV1001, neurodegeneration, anti-aging, Alzheimer’s disease

## Abstract

GV1001, which mimics the activity of human telomerase reverse transcriptase, protects neural cells from amyloid beta (Aβ) toxicity and other stressors through extra-telomeric function, as noted in our prior *in vitro* studies. As per a recent phase II clinical trial, it improves cognitive function in patients with moderate to severe dementia. However, the underlying protective mechanisms remain unclear. This study aimed to investigate the effects of GV1001 on neurodegeneration, senescence, and survival in triple transgenic Alzheimer’s disease (3xTg-AD) mice. GV1001 (1 mg/kg) was subcutaneously injected into old 3xTg-AD mice thrice a week until the endpoint for sacrifice, and survival was analysed. Magnetic resonance imaging (MRI) and Prussian blue staining (PBS) were performed to evaluate entry of GV1001 entrance into the brain. Diverse molecular studies were performed to investigate the effect of GV1001 on neurodegeneration and cellular senescence in AD model mice, with a particular focus on BACE, amyloid beta_1-42_ (Aβ_1-42_), phosphorylated tau, volume of dentate gyrus, β-galactosidase positive cells, telomere length, telomerase activity, and ageing-associated proteins. GV1001 crossed the blood-brain barrier, as confirmed by assessing the status of ferrocenecarboxylic acid-conjugated GV1001 using magnetic resonance imaging and PBS. GV1001 increased the survival of 3xTg-AD mice. It decreased BACE and Aβ_1-42_ levels, neurodegeneration (i.e., reduced CA1, CA3 and dentate gyrus volume, decreased levels of senescence-associated β-galactosidase positive cells, and increased telomere length and telomerase activity), and levels of ageing-associated proteins. We suggest that GV1001 exerts anti-ageing effects in 3xTg-AD mice by reducing neurodegeneration and senescence, which contributes to improved survival.

## INTRODUCTION

Alzheimer’s disease (AD) is one of the most critical neurodegenerative disorders, as elderly people over 65 years of age are increasing worldwide [1–3]. AD is a major burden for aging societies worldwide, and its treatment should be established as soon as possible for the future of humans. Although there have been many studies on the development of AD treatment, there is no definitive cure, except for symptomatic treatment with donepezil, rivastigmine, galantamine, and memantine. Antibodies against amyloid beta (Aβ) have been under clinical trials and have given us hope [4, 5]. For example, aducanumab was approved by the Food and Drug Administration in the United States in June 2021, although its efficacy remains controversial. Despite the use of various antibodies to treat AD, there are still many unmet medical needs.

Aging is an inevitable and definite risk factor for AD, which may accelerate aging [6]. Aging, which is eventually associated with progressive physical deterioration and increased vulnerability to death, cannot be avoided [7]. In contrast to chronological aging, physical aging, which is more important for health and aging-related disorders, may differ between individuals. Physical aging results in processes such as genomic instability, telomere attrition, epigenetic alterations, loss of proteostasis, mitochondrial dysfunction, cellular senescence, deregulated nutrient sensing, stem cell exhaustion, and altered intercellular communication [8], and vice versa. The aging process can increase the activity of β-site amyloid precursor protein cleaving enzyme (β-secretase, BACE), which contributes to the amyloidogenic pathogenesis of AD, and can cause the loss of neurons and synapses in the cerebral cortex, which are the main characteristics of AD [9–11]. Telomere shortening due to aging has also been reported to be closely related to cognitive decline and AD [12, 13]. Due to the importance of the aging process in the pathogenesis of AD [6], anti-aging therapy has been regarded as a possible and valuable treatment for AD. Although there have been many clinical trials using drugs with anti-aging effects for AD, none have shown clinical efficacy and have been approved for clinical use. However, as anti-aging is still considered one of the most fascinating treatments for AD, notable effort has been dedicated to the development of new therapeutic strategies with anti-aging effects.

GV1001 is a small peptide containing 16 amino acids that mimics a fragment of the active catalytic site of human telomerase reverse transcriptase (hTERT). GV1001 has been previously reported to have various biological functions, including antioxidant, anti-inflammatory, anti-aging, anti-apoptotic, and mitochondrial stabilisation effects, mimicking the extra-telomeric function of hTERT [14–16]. Based on these beneficial effects, GV1001 was applied in a phase II clinical trial for the treatment of moderate to severe AD, and the results showed that a significant improvement in cognitive function was observed in patients with moderate to severe AD treated with GV1001, at weeks 12 and 24 [17]. Despite these beneficial effects of GV1001 in an *in vitro* model of AD and in patients with moderate to severe dementia, the exact *in vivo* mechanisms of action are unclear.

Therefore, we hypothesised that GV1001 might have anti-aging effects and improve neurodegeneration and senescence *in vivo* as a possible mechanism for its beneficial effects on AD. To test these hypotheses, we conducted this study to determine whether GV1001 could increase the survival of triple transgenic Alzheimer’s disease (3xTg-AD) mice. In addition, various molecular experiments to analyse the level of BACE, level of amyloid beta_1-42_ (Aβ_1-42_), volume of the hippocampus and cortex, abundance of senescence-associated β-galactosidase-positive cells, telomere length, telomerase activity, and levels of aging-associated proteins were performed to demonstrate the anti-aging effects of GV1001 on neurodegeneration and senescence in 3xTg-AD mice.

## RESULTS

### GV1001 enters the brain through the blood-brain barrier (BBB)

**Figure 1:**
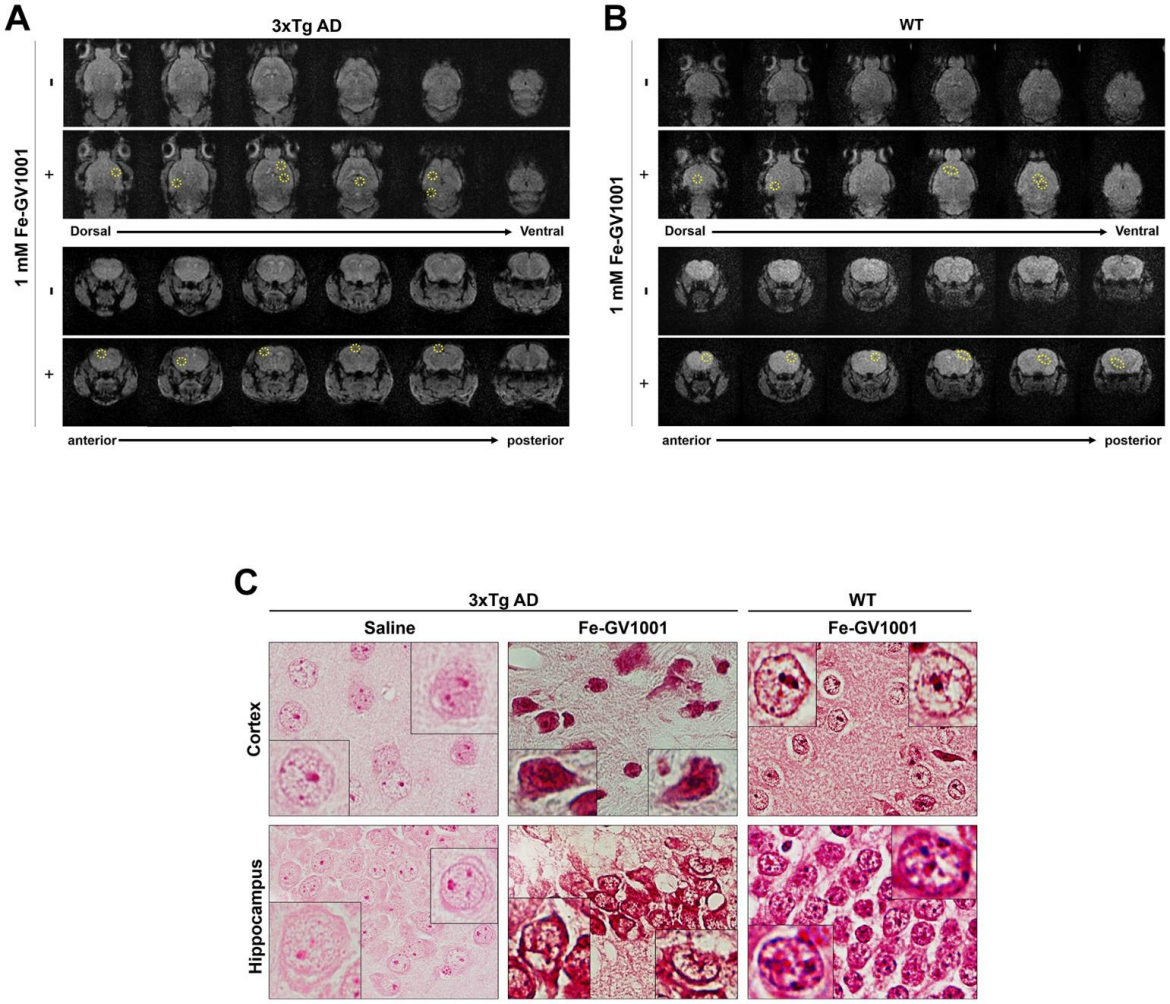
**Penetrance of GV1001 into the brain through the blood-brain barrier.** (**A**, **B**) We performed magnetic resonance imaging (MRI) to determine whether GV1001 could enter the brain. Ferrocenecarboxylic acid (Fe)-GV1001 (1 mg/kg) was subcutaneously injected into 3xTg-AD (12-month-old) and wild-type (WT) (8-month-old) mice. After 1 h, two-dimensional (2D) axial (Axl) fast field echo (FFE) and 2D coronal (Cor) FFE images were acquired using 3 T MRI. The entire brain was observed in several sections in the dorsoventral and anteroposterior planes using 2D Axl FFE and 2D Cor FFE images. This result shows that GV1001, represented by dark signals, entered the brains of both 3xTg-AD and WT mice (yellow circles in A and B, respectively). (**C**) Prussian blue staining.

To confirm that GV1001 can enter the brain through the BBB, 200 μl of 1 mM ferrocenecarboxylic acid-conjugated GV1001 (Fe-GV1001), which has been verified to enter diverse stem cells very efficiently and can be used to track transplanted stem cells in the brain [18], was injected into the tail vein of 3xTg-AD (B6:129-Psen1tm1Mpm Tg [APPSwe, tauP301L] 1Lfa/Mmjax) and WT mice. Before and after injection, MRI was performed as previously described. MR images showed dots with low signal intensity in the brains (surrounded by yellow circles) of 3xTg AD mice (Figure 1A) and WT mice (Figure 1B) after injection (+ in the figures) compared to before it (- in the figures). These dots were confirmed to be Fe-GV1001 via Prussian blue staining (Figure 1C), and these findings showed that Fe-GV1001 entered the brain across the BBB.

### GV1001 significantly improves the survival of old-aged 3xTg AD mice

**Figure 2:**
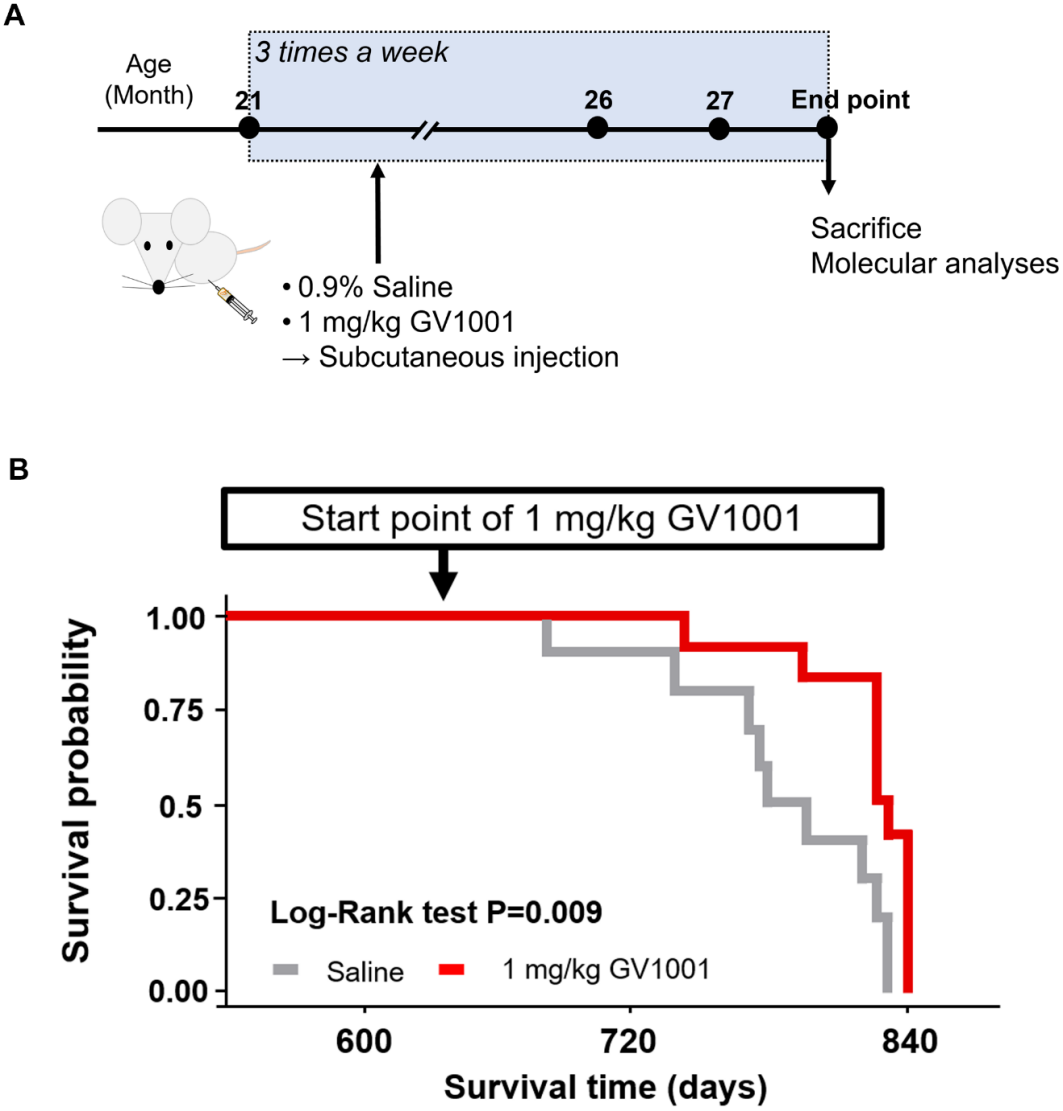
**Survival of old 3xTg-AD mice after GV1001 treatment.** (**A**) 1 mg/kg of GV1001 or an equivalent volume of 0.9% saline was subcutaneously injected into old-aged 3xTg-AD mice (n = 10 [male =5 and female 5]; control, n = 12 [male = 7 and female = 5]; 1 mg/kg of GV1001) from the age of 21 months until the mice were considered ready to sacrifice (endpoint). Injections were administered thrice a week until the endpoint. (**B**) Survival curves plotted using the Kaplan-Meier estimator. The survival of mice was markedly prolonged by administration of 1 mg/kg.

Survival after starting GV1001 treatment at 21 months was evaluated until the endpoint (Figure 2A), which was determined based on the criteria for an appropriate endpoint according to the CCAC guidelines (Supplementary Tables 1, 2), to assess the effect of GV1001 on the lifespan of old-aged 3xTg AD mice corresponding to advanced AD [19]. Survival probability analysis showed that GV1001 treatment markedly improved the survival of old-aged 3x Tg AD mice (*p* = 0.009, Figure 2B).

### GV1001 reduces Aβ_1-42_ by decreasing BACE and phosphorylated tau (p-tau) in old-aged 3xTg AD mice

**Figure 3:**
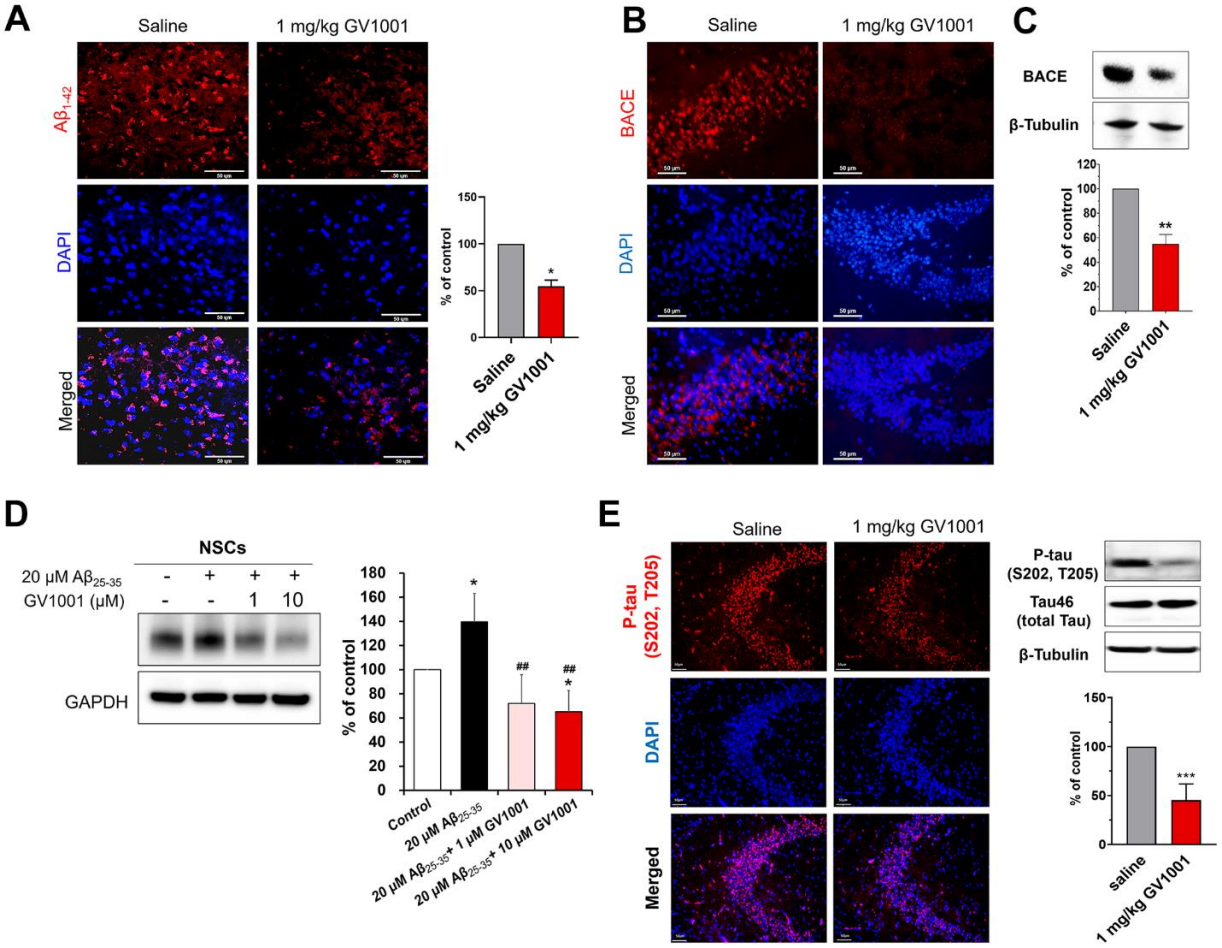
**Effect of GV1001 on amyloid beta, β-secretase (BACE) and p-tau levels.** (**A**) GV1001 significantly ameliorated the levels of Aβ_1-42_ in the brains of aged 3xTg-AD mice. (**B**, **C**) Expression of BACE was downregulated in old 3xTg-AD mice after treatment with 1 mg/kg GV1001, as shown by western blotting (**B**) and immunohistochemistry (**C**). (**D**) NSCs were isolated from embryonic rodent brains, cultured, and expanded. Then, they were treated with different concentrations of GV1001 (0, 1, or 10 μM) and 20 μM Aβ_25-35_ for 48 h. The expression of BACE was markedly upregulated in NSCs following treatment with 20 μM Aβ_25-35_, although treatment with 1 or 10 μM GV1001 significantly downregulated the expression of BACE. (**E**) GV1001 significantly reduced the levels of p-tau in the hippocampus (CA3). Data are expressed as the mean (% of control) ± standard deviation of three to five independent experiments. The treatment groups were compared using Tukey’s post-hoc test after one-way or two-way ANOVA. *p < 0.05; **p < 0.01 (vs. the control group), ^##^p < 0.01 (vs. NSCs treated only with 20 μM Aβ). Scale bar = 50 μm.

Diverse molecular studies have been performed to explain the positive effects of GV1001 on the survival of old-aged 3xTg AD mice. The effect of GV1001 on Aβ_1-42_ expression was investigated using immunohistochemistry (Figure 3A). Treatment with 1 mg/kg of GV1001 noticeably diminished the expression of Aβ_1-42_ in the brains of 3xTg AD mice compared with that of saline-treated mice (Figure 3A and Supplementary Figure 1A). BACE is a key enzyme in Aβ_1-42_ production; therefore, the expression of BACE was also evaluated using western blotting and immunohistochemistry. The results showed that 1 mg/kg of GV1001 significantly decreased BACE expression (Figure 3B, 3C). To double-check the effect of GV1001 on BACE expression, NSCs were treated with 20 μM Aβ_25-35_ and/or different doses of GV1001 (1 and 10 μM) *in vitro*, and BACE expression was considerably reduced in NSCs treated with GV1001 (Figure 3D). In addition, treatment with 1 mg/kg of GV1001 reduced the expression of p-tau (S202. T205) in the hippocampus of 3xTg AD mice (Figure 3E and Supplementary Figure 1B).

### GV1001 markedly inhibited neurodegeneration and senescence of the hippocampus in old-aged 3xTg AD mice

**Figure 4:**
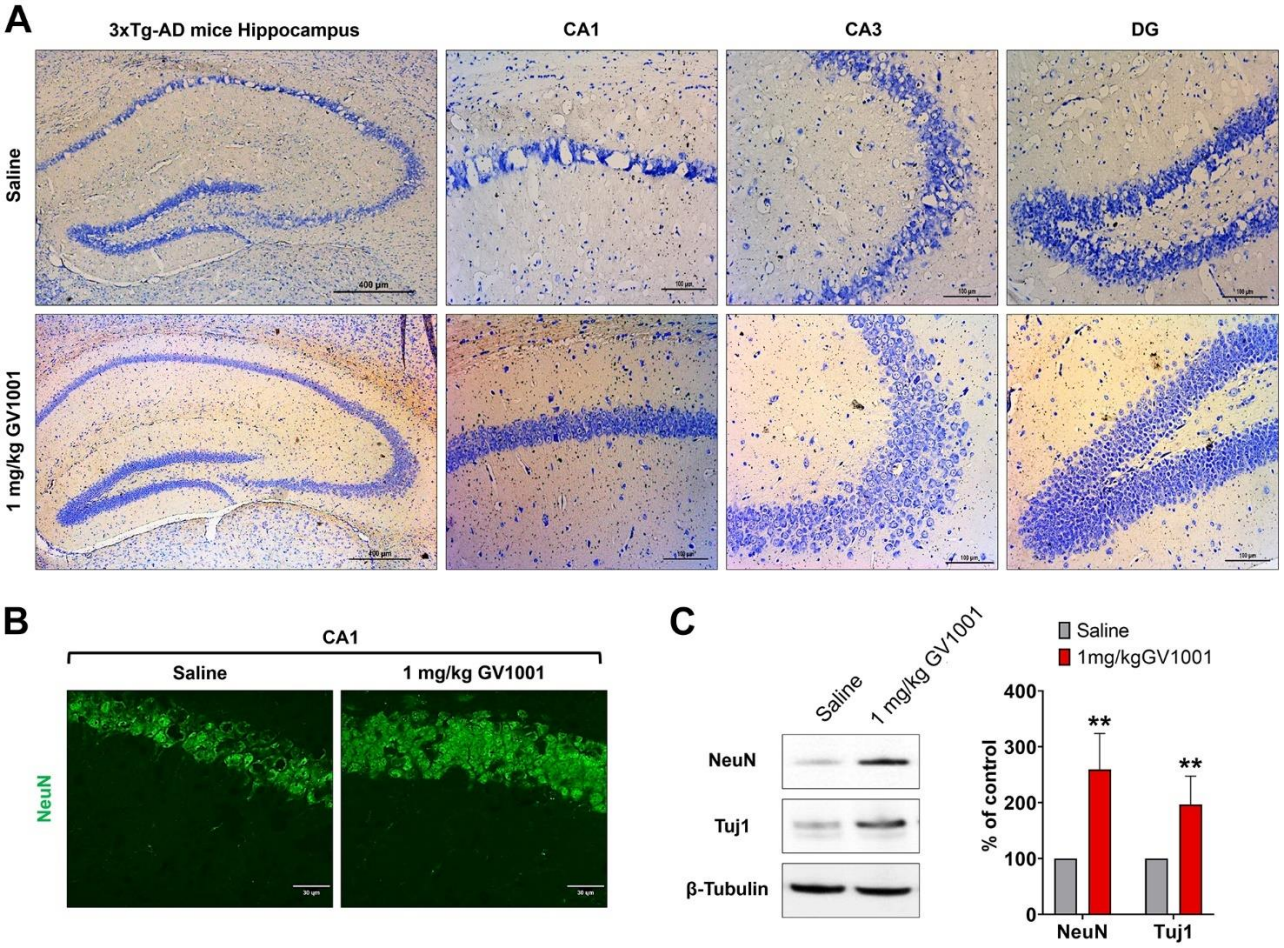
**Effect of GV1001 on neurodegeneration.** Markers of neurodegeneration were studied in aged 3xTg-AD mice treated with GV1001 and saline. (**A**) Neurodegeneration was evaluated by the volume of the CA1, CA3 and dentate gyrus (DG) in the hippocampus and (**B**, **C**) expression of neuronal markers, such as neuronal nuclei (NeuN) and neuron-specific β III tubulin (Tuj1) of the CA1 in the hippocampus. GV1001 markedly prevented neuronal loss in the hippocampus of 3xTg-AD mice. *P < 0.05; **P < 0.01 (vs. the control group). Scale bar = 30 μm (a) and 100 μm (b).

Nissl staining showed that neurodegeneration of the hippocampus was dominant in old-aged 3xTg AD mice, but treatment with 1 mg/kg of GV1001 markedly inhibited neurodegeneration (Figure 4A). The same finding was also found in immunohistochemistry using an anti-NeuN antibody specific for neurons (Figure 4B). Western blotting of the hippocampus of old-aged 3xTg AD mice showed that treatment with 1 mg/kg of GV1001 extensively increased the expression of NeuN and Tuj1, which are neuronal markers (Figure 4C), which is consistent with the results of nissl staining and IHC.

### Effect of GV1001 against neurodegeneration and senescence in the brain of old-aged 3xTg AD mice was achieved through anti-senescence activity

**Figure 5:**
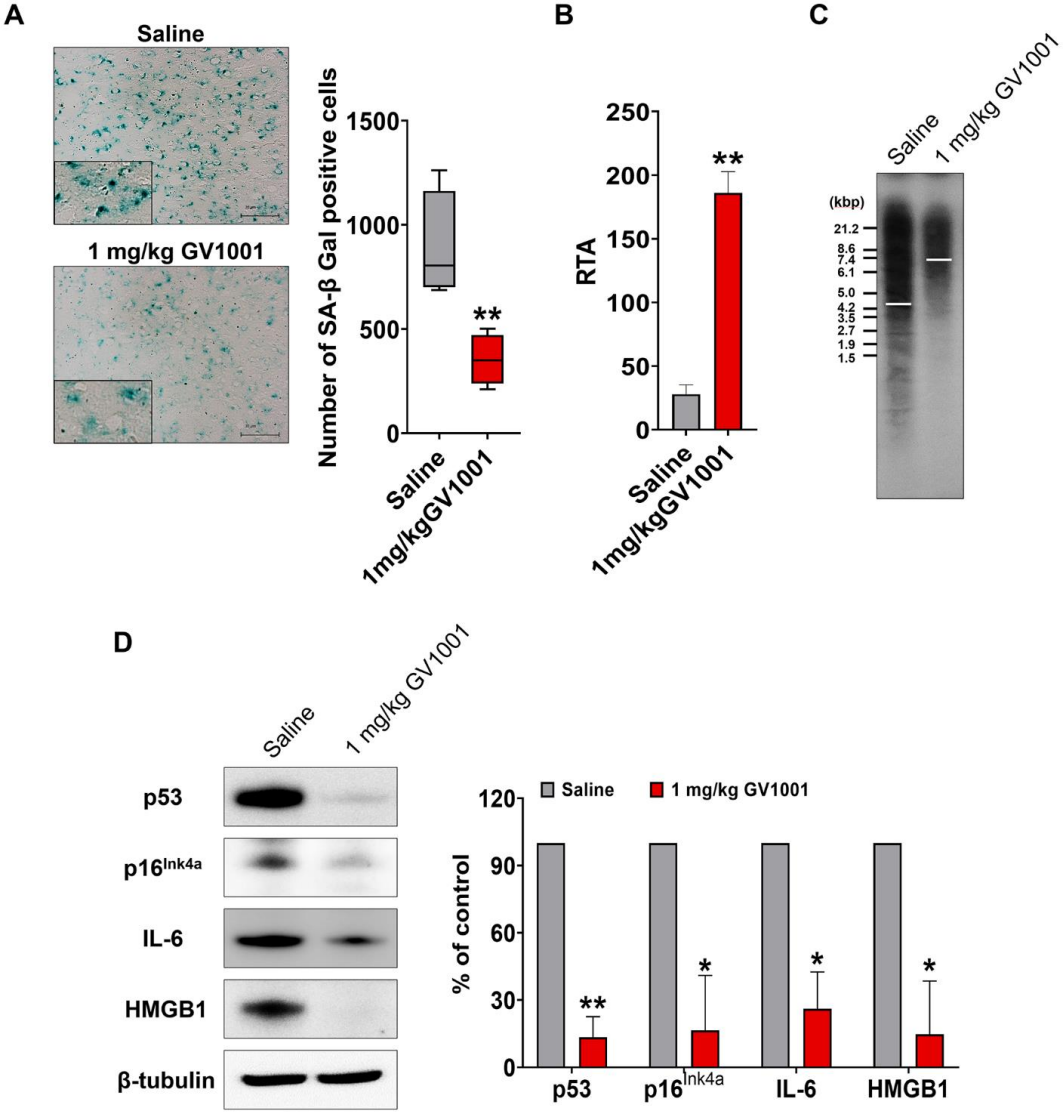
**Effect of GV1001 on senescence.** Markers of senescence were studied in middle-aged and old 3xTg-AD mice treated with GV1001 or saline. (**A**) GV1001 markedly reduced the expression of senescence-associated β-galactosidase, a well-known senescence marker, in old-aged 3xTg-AD mice. (**B**, **C**) Telomerase activity and telomere length were evaluated as senescence markers. GV1001 significantly increased both parameters in old-aged 3xTg-AD mice (B and C, respectively). (**D**) The expression of senescence markers, such as p53, p16^Ink4a^, interleukin-6, and high mobility group protein B1, in the brains of old 3xTg-AD mice treated with GV1001 and saline, was assessed, and the results showed that the expression levels of all senescence markers decreased in old-aged 3xTg-AD mice after treatment with GV1001. *P < 0.05; **P < 0.01 (vs. the control group). Scale bar = 30 μm (a, b).

Markers used to predict cell senescence, such as SA-β-Gal, telomerase activity, telomere length, p53, p16^Ink4a^, IL-6, and HMGB1, were measured in the brains of old-aged 3xTg AD mice. The results showed that SA-β Gal-positive cells were significantly reduced by treatment with 1 mg/kg GV1001 compared to saline treatment (Figure 5A). Telomerase activity, which is essential to maintain telomere length, and telomere length, which is well known to decrease with senescence of cells, were improved in the brains of old-aged 3xTg AD mice treated with 1 mg/kg of GV1001 (Figure 5B, 5C). Intracellular markers of senescence, such as p53, p16^Ink4a^, IL-6, and HMGB1, were considerably decreased in the brains of old-aged 3xTg AD mice treated with 1 mg/kg of GV1001 (Figure 5D). These results suggest that the effect of GV1001 on neurodegeneration in the brains of old-aged 3xTg AD mice may be related to its anti-senescence activity.

### Bioinformatics using the results of proteomics and RNA sequencing also suggests that GV1001 has the anti-senescence effect

**Figure 6:**
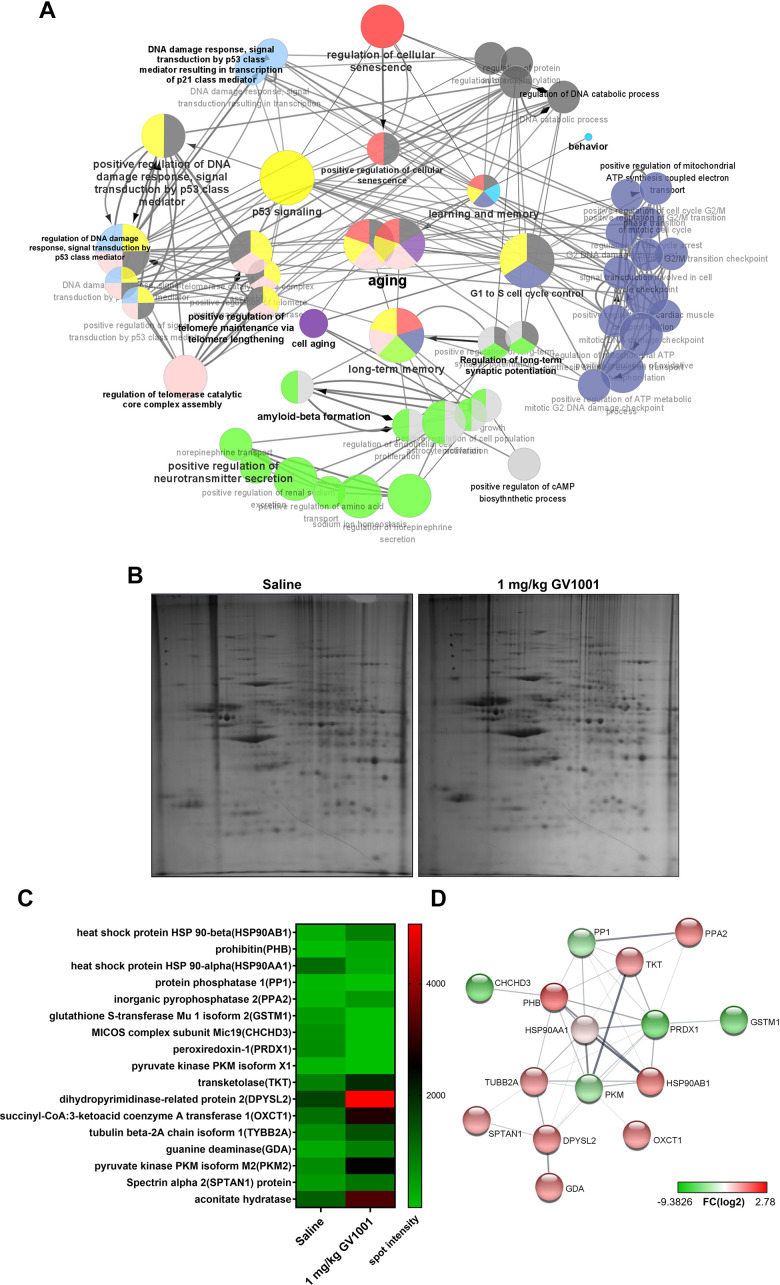
**Effect of GV1001 on aging, telomere maintenance, and long-term memory confirmed via bioinformatics analysis.** (**A**) ClueGO analysis visualised the significant functional enrichment results of upregulated and downregulated mRNAs associated with inflammation in old-aged 3xTg-AD mice treated with GV1001 compared to those treated with saline, using the Gene Ontology (GO) functional network. mRNAs that show significant differential expression are selected for analyses based on the following criteria: more than three-fold change, normalised data (log2) >4, and p < 0.05. The functionally grouped networks of the gene regulatory network and the target genes of these hub nodes were transferred to ClueGO and grouped as functional clusters. All nodes have been linked based on their kappa scores (≥0.3). Only the most significant term per group is labelled in bold, and their colours represent the functional groups to which they belong. Node size represents enrichment significance. (**B**) Proteomics show that GV1001 affected the levels of various intracellular proteins in the brains of 3xTg AD mice. Two-dimensional (2D) gel electrophoresis images. The first dimension is run using a 13-cm IPG strip with a pH gradient from 4 to 10. Coomassie blue staining is used to identify 24 proteins that increased or decreased more than two-fold. (**C**) Heat map shows protein levels. Protein expression values are log2-transformed. Red indicates a high expression level; green indicates a low expression level. (**D**) STRING analysis (functional protein association networks database, http://string-db.org/) of upregulated and downregulated proteins. Proteins are clustered based on the STRING evidence score. PPA2, inorganic pyrophosphatase 1; GSTM1, glutathione S-transferase Mu 1 isoform 2; MICOS, mitochondrial contact site and cristae organising system; CHCHD3, complex subunit Mic19; PRDX1, peroxiredoxin-1; DPYSL2, dihydropyrimidinase-related protein 2; OXCT1, succinyl-CoA:3-ketoacid coenzyme A transferase 1; TYBB2A, tubulin beta-2A chain isoform 1; SPTAN1, spectrin alpha 2; PHB, prohibitin; TKT, transketolaseHSP90AA1, HSP90AA1, heat shock protein 90 alpha class A member 1; HSP90AB1, heat shock protein 90 alpha class B member 1.

A messenger ribonucleic acid (mRNA) sequencing was performed to evaluate the effect of GV1001 on the expression of mRNAs in the brains of 3xTg AD mice, and the results were analysed using ClueGO. The significant functional enrichment results of the upregulated and downregulated mRNAs after GV1001 treatment were visualised using ClueGO (Figure 6A). An interesting finding of the GO analysis was the detection of critical pathways and functions, such as the regulation of cellular senescence, p53 signalling, positive regulation of telomere maintenance through telomere lengthening, long-term memory, positive regulation of cAMP biosynthesis, and aging. ClueGO analysis showed that GV1001 strongly affected the levels of senescence-related mRNAs.

Proteomics also confirmed that this effect of GV1001 in the brains of 3xTg AD mice was achieved through diverse intracellular signalling pathways (Figure 6B–6D). The heat map shows protein levels (Figure 6B). Protein expression values were log2-transformed. Red indicates a high expression level, and green indicates a low expression level (Figure 6C). The survival-related proteins including prohibitin [20, 21], dihyropyridinase-realated protein 2 (DPYSL2) [22], transketolase [23], pyruvate kinase isoform M2 (PKM2) [24], spectrin alpha 2 (spna2) [25] and aconitate hydratase [26] were increased with GV1001 treatment. STRING analysis showed up- and downregulated protein–protein interaction networks. Upregulated and downregulated proteins were clustered based on the STRING evidence score (Figure 6D).

## DISCUSSION

In the present study, we confirmed that GV1001 well enters the brain crossing the blood-brain barrier (Figure 1) and significantly prolongs the lifespan of AD transgenic mice (Figure 2) through diverse antiaging effects. As detailed evidence on GV1001’s anti-aging effects, GV1001 reduced levels of Aβ_1-42_ (Figure 3A) and BACE (β-secretase) (Figure 3B–3D) and it also restored volume of CA1, CA3 and dentate gyrus and increased neuronal markers in the hippocampus (Figure 4). Senescence-associated β-galactosidase positive cells and intracellular signaling proteins associated with aging were markedly decreased with GV1001 treatment (Figure 5A). In addition, GV1001 enhanced telomerase activity (Figure 5B) and increased telomere length (Figure 5C). RNA-seq and proteomics also confirmed that GV1001 inhibited aging-related signaling pathways and boosted anti-aging-associated ones (Figure 6).

Aging is an undeniable risk factor for AD and it leads to many changes like genomic instability, telomere attrition, epigenetic alterations, loss of proteostasis, mitochondrial dysfunction, cellular senescence, deregulated nutrient sensing, stem cell exhaustion, and altered intercellular communication [8], all of which are also well-established pathomechanism of AD. It is well known that level of BACE and senescence-associated β-galactosidase positive cells increases [10, 27] and telomere length and telomerase activity decreases [28] with aging. These are also related to the progression of AD. BACE and Aβ_1-42_ are related to amyloidogenic process after processing amyloid precursor protein (APP) [11]. Reducing the level of BACE and Aβ_1-42_ by GV1001 in 3xTg-AD mice, found in this study, indicates the possibility of GV1001 to cut down on amyloidogenic pathogenesis of AD by ameliorating initiation of Aβ formation.

Senescence-associated β-galactosidase positive cells and telomere attrition are well-known biomarkers for aging [27, 29]. Increased activity of β-galactosidase, which suggests the accelerated aging, is also found in AD [30]. Telomere length, a predictor of the future life of cells, progressively shortens with each cell division and aging process and it can be reversed by the telomerase [28, 31]. Telomerase is known to maintain telomere structure and decreased telomerase activity affects telomere shortening and cellular senescence [28, 32]. Shortening of telomere can also occur with aging of organism and shorter telomere length is linked to accelerated aging [31, 33]. Both shortening of telomere length and progression of AD have been reported to be associated with oxidative stress and inflammation [34, 35]. Shorter telomere length is known to be correlated with the mortality of general population and the risk of AD [13, 36]. In this study, GV1001 efficiently reduced β-galactosidase-positive cells and senescene-associated proteins and increased telomere length and telomerase activity in 3xTg-AD mice. These results represent GV1001 has anti-aging effects against accelerated aging in AD mice and can explain the increment of survival of them.

The expression of survival-related proteins, including prohibitin (PHB) [20, 21], dihyropyridinase-related protein 2 (DPYSL2) [22], transketolase (TKT) [23], pyruvate kinase isoform M2 (PKM2) [24], spectrin alpha 2 (SPTAN1) [25], and aconitate hydratase [26], increased with GV1001 treatment. According to proteomics, the levels of survival-related proteins, such as PHB, TKT, DPYSL2, PKM2, SPTAN1, and aconitate hydratase, increased after treatment with GV1001. PHB is known to affect mitochondrial membrane composition and its functionality [20]. Loss of PHB impairs mitochondrial architecture and leads to tau hyperphosphorylation and neurodegeneration [21]. TKT regulates the proliferation of hippocampal progenitor cells, and attenuation of TKT activity inhibits their proliferation [23]. PKM2 is found in the brain and liver and is involved in the repair and regeneration of these tissues [24]. SPTAN1 is a spectrin family protein expressed in neurons and glia and plays a role in stabilising the cytoskeleton [25]. Levels of aconitate hydratase, which is involved in energy generation and antioxidant systems, decrease with age [26, 37]. In conclusion, increasing the levels of the proteins described above might have a protective effect against neurodegeneration. Currently available treatments for AD (acetylcholinesterase inhibitors and N-methyl-D-aspartic acid receptor antagonists) have beneficial effects on cognition, function, behaviour, and clinical global changes in patients with AD [38]. However, they are not considered disease-modifying treatments that alter the underlying pathomechanism of AD [39]. According to a recent randomised controlled clinical trial, treatment with GV1001 improved cognitive function in patients with moderate to severe dementia compared to placebo treatment [17]. In addition to the previously confirmed effects of GV1001 (antioxidant, anti-inflammatory, anti-aging, antiapoptotic, and mitochondrial stabilisation effects) [14, 40], the neuroprotective effect confirmed in the current study (improving survival, reducing BACE and Aβ_1-42_, reducing atrophy of the dentate gyrus, and improving senescence in 3xTg-AD mice) explains the mechanisms of GV1001. GV1001 targets multiple processes of AD and may be a good candidate for disease-modifying treatments for AD.

Considering our previous *in vitro* findings [14, 15, 18], the beneficial effects of GV1001 are suggested to be related to its extra-telomeric function. The extra-telomeric function of hTERT affects various cellular processes, such as metabolism, stress response, epigenetic regulation, and mitochondrial function. Extra-telomeric function contributes to the regulation of chromatin structure, gene expression, DNA damage response, and interaction with various signalling pathways in the nucleus; it controls stress particles under non-stressed conditions, interacts with signalling pathways in the cytoplasm, reduces mitochondrial ROS and apoptosis, protects mitochondrial DNA, improves respiration, and increases complex 1 activity in mitochondria [41, 42]. All these effects on the extra-telomeric function of hTERT coincide with the most important changes during the anti-aging process [43, 44]. A recent study also suggested that increasing TERT with telomerase-increasing compounds protected hippocampal neurons from Aβ toxicity by enhancing the gene expression of neuronal survival and plasticity [39]. Also, GV1001 and gonadotropin-releasing hormone receptors (GnRHRs) were highly colocalized and GV1001 activated downstream of GnRHRs [45]. Beneficial effects of GV1001 were blocked after knockdown of GnRHRs [45]. It is reasonable to believe that the anti-aging effects of GV1001 in 3xTg-AD mice demonstrated in this study are also associated with extra-telomeric function and activating GnRHRs as an important mode of action against AD.

Some limitations of this study must be considered. First, animal models are of familial AD and cannot reflect the heterogeneous nature of humans enrolled in clinical trials [46]. The results of this study cannot explain all protective effects of GV1001. A more diverse protective mechanism of GV1001 in AD, including its anti-inflammatory effects, needs to be confirmed *in vivo*.

In conclusion, accelerated aging and AD are closely related, and this study confirmed that GV1001 has multiple anti-aging effects. These effects, including reducing neurodegeneration, BACE, Aβ_1-42_, atrophy of the dentate gyrus, and senescence, may improve the survival of 3xTg-AD mice and serve as evidence to explain the positive results shown in a clinical trial for the treatment of moderate to severe AD.

## MATERIALS AND METHODS

### Animals

Every effort was made to minimise the number of animals used and limit animal suffering. Each animal was used once. Old-aged 3xTg-AD mice (B6; 129-Psen1 tm1Mpm Tg [APPSwe, tauP301L] 1Lfa/Mmjax) and corresponding wild-type (WT) (B6129SF2/J) mice used in the experiments were obtained from Jackson Laboratory (Bar Harbour, ME, USA).

In this study, experimental 3xTg-AD mice (21 months old) were divided into two groups, with 12 animals assigned to each group. The experimental groups were as follows: 1) 0.9% saline and 2) 1 mg/kg of GV1001 (the most effective dose confirmed in another middle-aged 3xTg-AD mice study, Supplementary Figures 1, 2). GV1001 or an equivalent volume of saline was subcutaneously injected into old-aged 3xTg-AD mice three times a week from the age of 21 months to the endpoint for sacrifice, identified according to the Canadian Council on Animal Care (CCAC) guidelines (Supplementary Tables 1, 2), in a blinded manner [47]. The survival curves of old-aged 3xTg-AD mice were generated using the Kaplan-Meier method.

### Magnetic resonance imaging (MRI)

To visualise GV1001 in the brains of 3xTg-AD and WT mice by MRI, ferrocenecarboxylic acid (Fe) was conjugated to GV1001. Two-dimensional (2D) imaging with axial (Axl) fast field echo (FFE) and 2D coronal (Cor) FFE MRI was performed using a DS Micro 47 coil (Philips, Cambridge, MA, USA) with a 3.0 T MRI device (Philips, 3.0 T Ingenia CX, The Netherlands). Mouse brains were imaged using 2D Axl FFE MRI [repetition time/time echo (TR/TE) = 596 ms/16 ms; number of signal averaging (NSA) = 10 ms; sense = 2; slide thickness/gap = 0.8/0 mm; matrix = 132 × 130; field of view (FOV) = 39 × 39 mm] and 2D Cor FFE MRI (TR/TE = 596/16 ms; NSA = 10 ms; sense = 2; slide thickness/gap = 0.7/0 mm; FOV = 39 × 39 mm) before the injection of GV1001. The 3xTg-AD and WT mice received intravenous injections of 10 mM Fe-GV1001 (diluted, 1:10; volume, 200 μL). After 1 h, 2D Axl FFE and 2D Cor FFE images were obtained. The entire brain was observed in several sections of the dorsoventral and anteroposterior (AP) planes. All animals were sacrificed for histological examination at the end of the MRI.

### Prussian blue staining (iron staining)

Brain tissue samples from 3xTg-AD and wild-type mice were fixed in 4% paraformaldehyde, embedded in paraffin, sectioned (thickness, 3 μm), and transferred to slides. The samples were incubated in ethanol and xylene for 3 min each for deparaffinization and washed three times with deionized water. Thereafter, the slides were incubated in 10 ml iron staining solution (equal volumes of potassium ferrocyanide and hydrochloric acid solution) for 3 min and washed with deionized water. The slides were then incubated in nuclear fast red solution for 5 min, washed with deionized water, and allowed to dry. The slides were then mounted with mounting solution and observed under an ultraviolet light microscope, (Olympus Bx53 microscope, Olympus, Tokyo, Japan).

### Immunofluorescence staining

Brain tissues from old (21 months old) and middle-aged (12 months old) 3xTg-AD mice were perfused with 0.9% sodium chloride (NaCl) and 4% paraformaldehyde for fixation. The fixed tissues and cells were sectioned using a cryostat, washed three times in 0.01 M phosphate-buffered saline (PBS), and permeabilised in 50% alcohol for 30 min. They were then incubated in 0.3% H_2_O_2_ in PBS for 20 min, followed by incubation with 10% foetal bovine serum (FBS; Gibco, Grand Island, NY, USA) in PBS for 60 min. Cells were incubated overnight in 2% normal serum in PBS containing the following primary antibodies: rabbit anti-beta amyloid 1-42 antibody (1:100, ab10148, Abcam, UK), rabbit anti-BACE (1:100, 5606, Cell Signalling, USA) and phospho-Tau (Ser202, Thr205) (1:100; MN1020; Invitrogen, USA), mouse anti-neuronal nuclei (NeuN) (1:100; MAB377, Millipore, USA).

The following day, cells were incubated for 60 min in 2% normal serum in PBS containing the following secondary antibodies: Alexa Fluor 488-conjugated goat anti-rabbit (Molecular Probes, Eugene, OR, USA), tetramethylrhodamine-conjugated anti-rabbit (Molecular Probes), Alexa Fluor 488-conjugated goat anti-rabbit (A11008, Invitrogen), Alexa Fluor 594-conjugated goat anti-rabbit immunoglobulin G (IgG) (H+L) (A11037, Thermo Fisher Scientific, Waltham, MA, USA), Alexa Fluor-594-conjugated cross-adsorbed donkey anti-goat IgG (H+L) (A11058, Invitrogen), and Alexa Fluor 350-conjugated cross-adsorbed donkey anti-goat IgG (H+L) (A21081, Invitrogen). Cells were washed several times with PBS and mounted on glass slides with a mounting medium containing 4′,6-diamidino-2-phenylindole (DAPI) (P36931, Invitrogen). Immunostained cells were observed using a confocal fluorescence microscope (K1-fluo, Nanoscope Systems Inc., Daejeon, Korea; DMIRB, Leica, Wetzlar, Germany) at the appropriate excitation wavelengths.

### Neural stem cell culture

Neural stem cells (NSCs) were isolated from embryonic rodent brains, cultured, and expanded. The NSC line was cultured as previously described [48–51]. Briefly, Sprague Dawley rat embryos were decapitated on embryonic day 13 (E13). The brains were rapidly removed and placed in a Petri dish half full of ice-cold Hank’s balanced salt solution (137 mM NaCl, 5.4 mM potassium chloride, 0.3 mM sodium phosphate dibasic, 0.4 mM potassium phosphate monobasic, 5.6 mM glucose, and 2.5 mM 4-(2-hydroxyethyl)-1-piperazineethanesulphonic acid; GIBCO BRL). Single cells were dissociated from the whole cerebral cortex, lateral ganglionic eminence, and ventral midbrains of foetal rats. The resulting cells were plated at a density of 2 × 10^4^ cells/cm^2^ in culture dishes precoated with poly-L-ornithine/fibronectin in Ca^2+^/Mg^2+^-free PBS (Gibco) and cultured in N2 medium (Dulbecco’s modified Eagle’s medium/nutrients mixture F-12, 25 mg/L insulin, 100 mg/L transferrin, 30 nM selenite, 100 μM putrescine, 20 nM progesterone, 0.2 mM ascorbic acid, 2 nM L-glutamine, 8.6 mM D(+) glucose, and 20 nM sodium bicarbonate; Sigma, St. Louis, MO, USA) supplemented with basic fibroblast growth factor (10 ng/mL; R&D Systems, Minneapolis, MN, USA). The cultures were maintained at 37° C in a humidified 5% CO_2_ atmosphere for 4–6 days.

### Western blotting

The levels of Aβ_1-42_, BACE, p-tau (phosphorylated at S202 and T205), NeuN, doublecortin (DCX), neuron-specific beta-III tubulin (Tuj1), tumour protein 5 (p53), cyclin-dependent kinase inhibitor 2A (CDKN2A/p16^INK4a^), interleukin-6 (IL-6), high mobility group box (HMGB1), β-tubulin and Glyceraldehyde-3-Phosphate dehydrogenase (GAPDH) were analysed using western blotting, immediately after treatment for 1 or 8 h and after sacrifice. The tissue and cells were harvested, washed once with ice-cold PBS, and resuspended in 1.0 mL of 1× cytosol extraction buffer mix containing dithiothreitol (DTT) and protease inhibitors. After 10 min of incubation on ice, the cell suspension was sonicated (Sonoplus) five to ten times on ice. The samples were centrifuged at 3,000 rpm and 4° C for 10 min, and the supernatants were centrifuged again at 13,000 rpm for 30 min. Samples containing equal amounts (30 μg) of protein were resolved using 10% sodium dodecyl sulphate-polyacrylamide gel electrophoresis (SDS-PAGE) and transferred to nitrocellulose membranes (Amersham Pharmacia Biotech, Buckinghamshire, UK). The membranes were blocked with 5% skim milk and incubated with specific primary antibodies. The following antibodies were used: anti-beta amyloid 1-42 antibody (1:1000, ab10148, Abcam), anti-BACE (1:1000, 5% BSA, Cell Signalling), phospho-Tau (Ser202, Thr205) (1:500; MN1020; Invitrogen), anti-NeuN (1:1000, 5% BSA, MAB377, Millipore), anti-DCX (1 μg/mL, 5% BSA, ab28941, Abcam), anti-Tuj1 (1:1000, 5% BSA, ab18207, Abcam), anti-p53 (1:1000, 5% BSA, 2524, Cell Signalling), anti-CDKN2A/p16^INK4a^ (1:2000, 5% BSA, ab211542, Abcam), anti-IL-6 (1:20, 5% BSA, ab7737, Abcam), anti-HMGB1 (1:1000, 5% BSA, 3935, Cell Signalling), anti-β-tubulin (1:2000, 5% BSA, 2146, Cell Signalling), and anti-GAPDH (1:1000, 5% BSA, Cell Signalling). The membranes were washed with Tris-buffered saline containing 0.1% Tween-20, processed using horseradish peroxidase (HRP)-conjugated anti-rabbit or anti-mouse secondary antibody (Jackson ImmunoResearch Laboratories, Inc., West Grove, PA, USA), and detected using the enhanced chemiluminescence method (GenDEPOT, Katy, TX, USA). The bands were quantified using an image analyser (ImageQuant LAS 4000, GE Healthcare, Little Chalfont, UK).

### Nissl body staining

Slices of the hippocampal CA1, CA3 and dentate gyrus (DG) region of the brains of 3xTg-AD mice were air-dried and soaked overnight in 1:1 alcohol:chloroform. The slices were then immersed in 0.1% cresyl violet solution (0.1 g cresyl violet acetate (c5042, Sigma, St. Louis, MO, USA) and 10 drops of glacial acetic acid (PHR1748, Sigma) for 10 min at 37° C. After incubation, slices were washed with distilled water and soaked in 95% ethyl alcohol for 30 min. Microscopic examination revealed the presence of distinct Nissl bodies. The slices were dehydrated with anhydrous alcohol, cleared in xylene, and mounted on glass slides using a mounting medium (Merck, Kenilworth, NJ, USA). The Nissl bodies were then observed under a Leica microscope (DM4000B, Leica Biosystems) to take 4× and 20× bright field images.

### Senescence-associated-β-galactosidase (SA-β-Gal) staining

The SA-β-Gal activity was measured using a β-gal staining kit (9860, Cell Signalling Technology) according to the manufacturer’s instructions. Briefly, fixed tissues were washed twice with PBS and stained with a β-gal staining solution containing 5-bromo-4-chloro-3-indolyl-b-D-galactopyranoside. Following overnight incubation at 37° C, blue-stained senescent cells were identified under a confocal fluorescence microscope (K1-Fluo, Nanoscope Systems Inc.).

### Telomerase activity

Telomerase activity was measured using the TeloTAGGG Telomerase PCR enzyme-linked immunosorbent assay (ELISA)^PLUS^ kit (Roche Applied Science, Penzberg, Germany) according to the manufacturer’s instructions. Cells were fluorimetrically assessed using an ELISA plate reader (Synergy H1 Hybrid Reader, BioTek Instruments, Winooski, VT, USA) at a wavelength of 450 nm and a reference wavelength of approximately 690 nm.

### Telomere length

DNA was extracted from cells and tissues using a High Pure PCR Template Preparation Kit (Roche Boehringer-Mannheim, Grenzach-Wyhlen, Germany). Telomere length analysis was performed using a nonradioactive TeloTAGGG telomere length assay (Roche Boehringer-Mannheim), as described by the manufacturer. Briefly, 2–4 μg of DNA from each sample was digested using Hinf I/RsaI enzyme mix and separated via gel electrophoresis. DNA fragments were transferred to a nylon membrane (GE Healthcare, Little Chalfont, UK) by Southern transfer and hybridised to a digoxigenin (DIG)-labelled probe specific for telomeric repeats. The membrane was incubated with a DIG-specific antibody conjugated to alkaline phosphatase, and the probe was visualised by chemiluminescence detection using an image analyser (ImageQuant LAS 4000, GE Healthcare). The mean telomeric repeat binding factor lengths were compared with those of the molecular weight standard.

### mRNA sequencing analysis

RNA sequencing of the hippocampal tissues from 3xTg-AD mice were performed by E-Biogen (Seoul, Korea).

### RNA isolation

Total RNA from the hippocampal tissues of 3xTg-AD mice was isolated using the TRIzol reagent (Invitrogen). RNA quality was assessed on an Agilent 2100 bioanalyzer using the RNA 6000 Nano Chip (Agilent Technologies, Amstelveen, The Netherlands), and RNA quantification was performed using an ND-2000 spectrophotometer (Thermo Fisher Scientific).

### Library preparation and sequencing

Libraries were prepared using 2 μg of total RNA using a SMARTer Stranded RNA-Seq Kit (Clontech Laboratories, Inc., Redwood City, CA, USA). mRNA enrichment was performed using a Poly(A) RNA Selection Kit (Lexogen, Inc., Vienna, Austria). The isolated mRNA was used for cDNA synthesis and shearing following the manufacturer’s instructions. Indexing was performed using Illumina indexes 1–12. The enrichment step was carried out using polymerase chain reaction (PCR). Subsequently, the libraries were checked using an Agilent 2100 bioanalyzer and DNA High Sensitivity Ki t (5067-4626, Agilent Technologies, Inc., Santa Clara, CA, USA) to evaluate the mean fragment size. Quantification was performed using the library quantification kit for the StepOne Real-Time PCR System (Life Technologies, Inc., Carlsbad, CA, USA). High-throughput sequencing was performed as sequencing of paired-end 100 base pairs using a HiSeq 2500 (Illumina, Inc., San Diego, CA, USA).

### Data analysis

mRNA-Seq reads were mapped using the TopHat software tool to obtain the alignment file [52]. Differentially expressed genes (DEGs) were determined based on counts from unique and multiple alignments using coverage in BEDTools [53]. The read count data were processed based on the quantile normalization method using an Edge R in R (R Development Core Team) and Bioconductor [54]. The alignment files were also used to assemble transcripts, estimate their abundance, and detect the differential expression of genes or isoforms using Cufflinks (http://cole-trapnell-lab.github.io/cufflinks/). We used the fragments per kilobase of exon per million fragments method to determine the expression levels of the gene regions. Gene classification was based on searches performed in the Database for Annotation, Visualization and Integrated Discovery (DAVID, https://david.ncifcrf.gov/), which is designed to provide tools for the functional interpretation of large lists of genes/proteins [55, 56].

### Gene ontology (GO) and signature analyses

GO analysis was conducted using DAVID bioinformatics resources [55]. The top 256 upregulated and downregulated DEGs were used for enrichment analysis using DAVID and the Cytoscape plug-ins ClueGO and CluePedia. The criteria to select significant DEGs for these analyses were folding change >3, normalised data (log_2_) >4, and P-value < 0.05. We used the ClueGO v2.5.8 app of Cytoscape v3.8.2 with global network specificity to visualise the significant functional enrichment results of our study [57]. Using ClueGO analysis, we identified upregulated and downregulated mRNAs, which were confirmed via RNA-Seq, in 3xTg-AD mice treated with GV1001 compared to those of 3xTg-AD mice treated with saline. Only the most significant term per group is labelled, and the colours represent the functional groups to which they belong. The size of the node represents the significance of enrichment. The functionally related groups partially overlapped.

### Proteomics

### Protein sample preparation

Cells were washed twice with ice-cold PBS and sonicated for 10 s with a Sonoplus homogeniser (Bandelin Electronics, Berlin, Germany) in a sample lysis solution composed of 7 M urea and 2 M thiourea, 4% 3-([3-cholamidopropyl] dimethylammonio)-1-propanesulphonate, 1% (w/v) DTT, 2% (v/v) Pharmalyte, and 1 mM benzamidine. The proteins were extracted for 1 h at 27° C using a vortex mixer. After centrifugation at 12,000 rpm for 1 h at 15° C, the insoluble material was discarded, and the soluble fraction was used for 2D gel electrophoresis. Protein concentrations were determined using the Bradford method.

### 2D PAGE

Immobilised pH gradient (IPG) dry strips (4-10 NL IPG, 13 cm, GE Healthcare) were reswelled for 9.5 h with Destreak rehydration solution and 0.5% IPG buffer and loaded with 150 μg of the sample. Isoelectric focusing (IEF) was performed at 20° C using an Ettan IPGphor 3 instrument (GE Healthcare, UK) following the manufacturer’s instructions. For the IEF, the voltage was linearly increased at 100–8,000 V over 7 h for sample entry and then maintained at 8,000 V. The focus was complete after 55 kVh. Before running the second dimension, the strips were incubated for 15 min in equilibration buffer (75 mM Tris-Cl, pH 8.8, containing 6 M urea, 2% SDS, 0.002% bromophenol blue stock solution, and 29.3% glycerol), first with 1% DTT and then with 2.5% iodoacetamide. Equilibrated strips were inserted into SDS-PAGE gels (13 × 18 cm, 12%). SDS-PAGE was performed using an SE600 2D system (GE Healthcare, UK) following the manufacturer’s instructions. The 2D gels were run at 20° C for 1,700 Vh.

### Image analysis

Quantitative analysis of digitised images was performed using ImageMasterTM 2D Platinum 7.0 software (GE Healthcare, UK) according to the protocols provided by the manufacturer. The intensity of each spot was normalised to the total valid spot intensity. The selected protein spots showed a difference in protein expression of at least two-fold compared with the control or normal samples.

### Peptide mass fingerprinting (PMF)

For protein identification with PMF, protein spots were excised, digested with trypsin (Promega, Madison, WI, USA), mixed with α cyano-4-hydroxycinnamic acid in 50% acetonitrile/0.1% trifluoroacetic acid (TFA), and subjected to matrix-assisted laser desorption ionisation-time of flight analysis (Microflex LRF 20, Bruker Daltonics, Billerica, MA, USA), as described by Fernandez et al. [58]. Spectra were collected from 300 shots per spectrum over the mass-to-charge (m/z) range of 600–3000 and calibrated by two-point internal calibration using trypsin autodigestion peaks (m/z 842.5099 and 2211.1046). The peak list was generated using Flex Analysis 3.0 (Bruker Daltonik GmbH, Bremen, Germany). The threshold used for peak selection was as follows: 500 for the minimum resolution of monoisotopic mass and 5 for single-to-noise (S/N). The search programme MASCOT, developed by Matrix Science (http://www.matrixscience.com/) [59], was used for protein identification using PMF. The following parameters were used for the database search: trypsin as the cleaving enzyme, a maximum of one missed cleavage, iodoacetamide (Cys) as a complete modification, oxidation (Met) as a partial modification, monoisotopic mass, and mass tolerance of ± 0.1 Da. The PMF acceptance criteria were based on probability scoring.

### Statistical analysis

All data are presented as mean ± standard deviation (SD) of five or more independent experiments. Statistical analyses of three or more datasets were performed using one-way analysis of variance (ANOVA), followed by Tukey’s post-hoc comparisons. Student’s *t*-test was used for comparison between the two groups. The survival outcomes were assessed using the Kaplan-Meier method and compared using log-rank tests. P-values <0.05 were considered statistically significant, and statistical analyses were performed with SPSS 21.0 (SPSS Inc., Chicago, IL, USA).

### Data and materials availability

The data and materials that support the findings of current study are available from the corresponding author upon reasonable request.

## Supplementary Material

Supplementary Figures

Supplementary Tables
