# Supplementary material for: GV1001 reduces neurodegeneration and prolongs lifespan in 3xTg-AD mouse model through anti-aging effects
Source: Aging (Albany NY). 2024 Jan 31;16(3):1983–2004. doi: 10.18632/aging.205489 (PMC10911355; doi:10.18632/aging.205489)
Supplement: Supplementary Figures [file aging-16-205489-s001.pdf]

SUPPLEMENTARY FIGURES

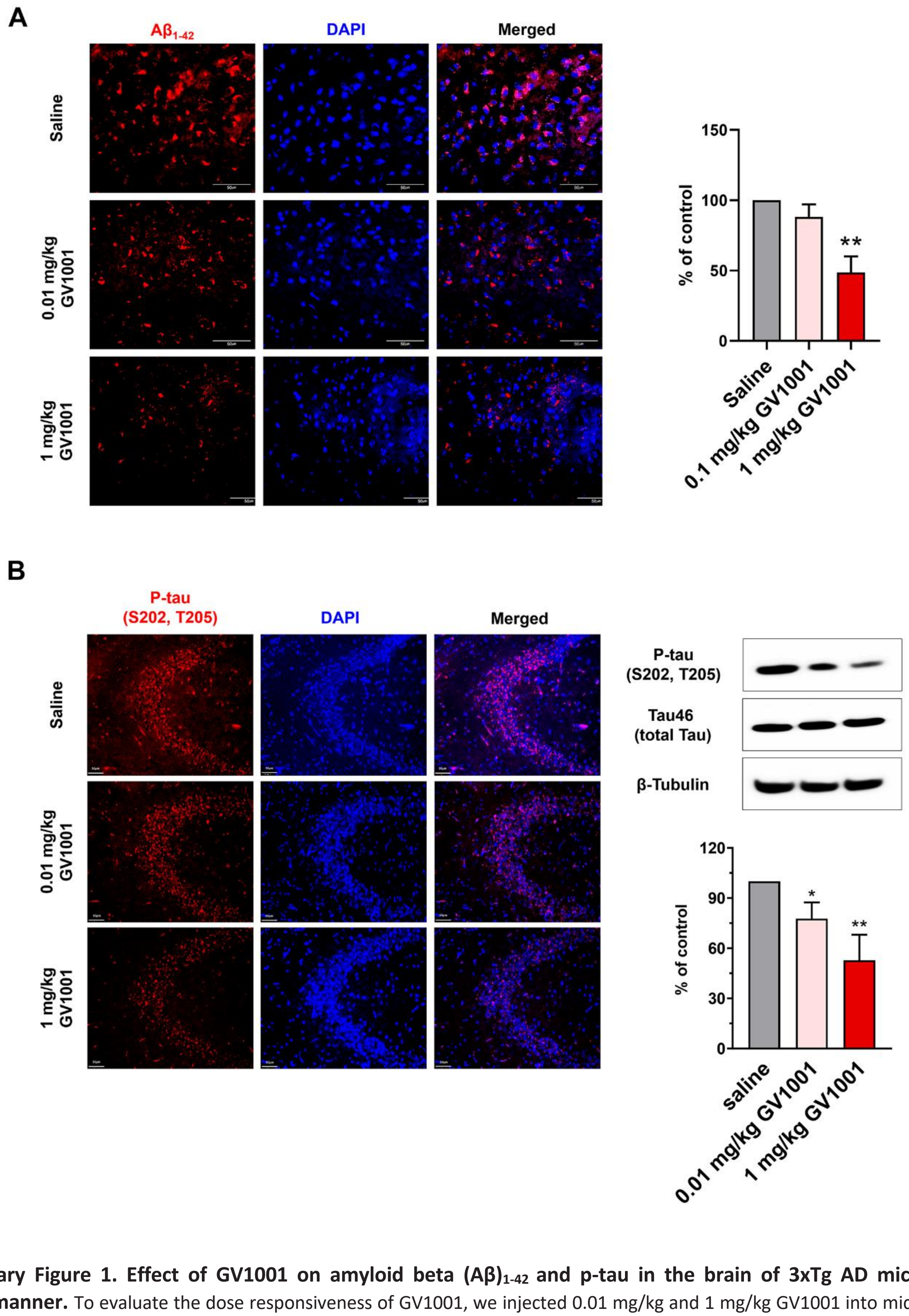

Supplementary Figure 1. Effect of GV1001 on amyloid beta (Aβ)$_{1-42}$ and p-tau in the brain of 3xTg AD mice in a dose-dependent manner. To evaluate the dose responsiveness of GV1001, we injected 0.01 mg/kg and 1 mg/kg GV1001 into middle-aged 3xTg AD mice subcutaneously for two months, from 12 months to 14 months of age. The results showed that 1 mg/kg GV1001 had a stronger effect against Aβ$_{1-42}$ (**A**) and p-tau (**B**) in the brains of middle-aged 3xTg mice than 0.01 mg/kg.

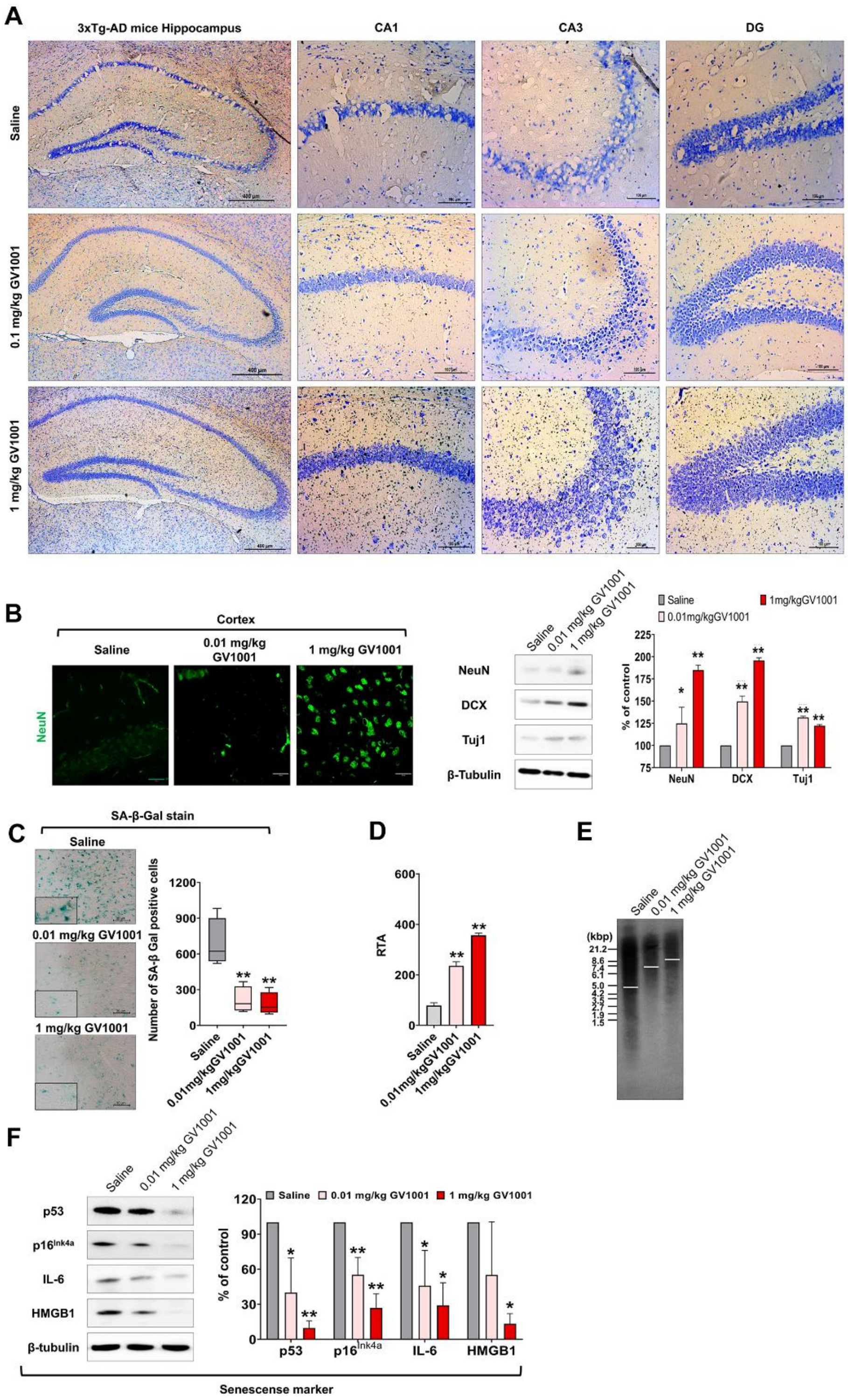

Supplementary Figure 2. GV1001 has a strong effect on senescence in the brain of 3xTg AD mice in a dose-dependent manner. Injection of 1 mg/kg GV1001 showed stronger effect than 0.01 mg/kg against neurodegeneration (**A**, **B**), and senescence (**C**–**F**) in the brains of middle-aged 3xTg AD mice. Therefore, we used 1 mg/kg GV1001 in the main study.
