# Supplementary material for: GV1001 reduces neurodegeneration and prolongs lifespan in 3xTg-AD mouse model through anti-aging effects
Source: Aging (Albany NY). 2024 Jan 31;16(3):1983–2004. doi: 10.18632/aging.205489 (PMC10911355; doi:10.18632/aging.205489)
Supplement: Supplementary Tables [file aging-16-205489-s002.pdf]

SUPPLEMENTARY TABLES

Supplementary Table 1. Criteria for an appropriate endpoint.

	Variable	Score
**Weight change**		
0	normal	
1	<10% Weight loss	
2	10~15% Weight loss	
3	>20% Weight loss	
**Body Condition Scoring, BCS (Extended Table. 2)**		
0	BCS >3	
1	BCS >2 and <3	
2	BCS >1 and <2	
3	BCS 1 or less	
**Appearance**		
0	normal	
1	lack of hair trim	
2	Rough hair, nose / eye discharge	
3	Severely rough hair, abnormal posture, dilated pupil	
**Clinical symptoms**		
0	normal	
1	Slight change	
2	1~2° C Body temperature change, heart rate and respiratory rate ~ 30% increase	
3	Changes in body temperature over 2° C, heart rate and respiratory rate ~ 50% increase or decrease abruptly.	
**behavior**		
0	normal	
1	Slight change	
2	Abnormal, decreased motion, decreased awareness, inactive	
3	Crying cry, self-cutting, hardly moving	
**Response to external reaction**		
0	normal	
1	Mild depression / increased reaction	
2	Severe abnormal reactions.	
3	Aggressive reaction or coma.	
	**Total**	

Various items were used to evaluate the condition of old 3xTg AD mice, as shown in Supplementary Table 1 [Establishing Humane Endpoints - Office of Research Ethics (https://www.compliance.iastate.edu.policies.docs)]. A total score of >5 or a score of 3 for any variable was determined, regardless of the total score for humane euthanasia.

Supplementary Table 2. Body condition score.

Mouse	
BC1<br>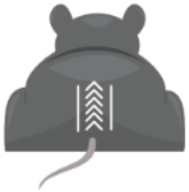	Haggard / Weakness<br>• The skeleton protrudes prominently. Almost no flesh.
BC2<br>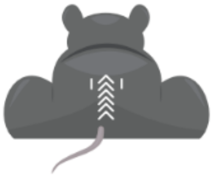	Bad management<br>• The separation of the protruding vertebra was observed.<br>• Pelvic bones of the dorsal can be easily palpated.
BC3<br>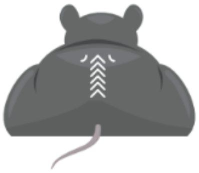	Proper management<br>• The spinal and pelvic bones are not noticeable, and can be palpable with slight pressure.
BC4<br>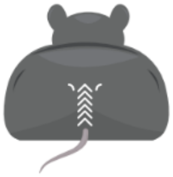	Excessive management<br>• Dorsal bones are not segmented but are continuously observed.<br>• The vertebrae must be reliably pressurized.
BC5<br>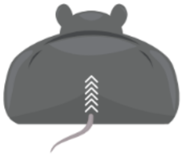	obesity<br>• The mouse seems to be flat and bulging.<br>• The skeleton has disappeared because the skin and subcutaneous fat are thick.

A body condition score (BCS) is used to evaluate fat and muscle development in livestock. Scoring techniques for mice are widely used to assess their health and fitness. BCS is an easy and objective assessment method that can be incorporated into humane endpoints. BCS can provide a more accurate estimate of health and fitness than body weight measurements, and it is useful in chronic studies in which animals lose muscle mass and fat over time.
